# Evaluating Surgical Trends and Outcomes in Stress Urinary Incontinence: A Clinical Audit at a District General Hospital

**DOI:** 10.7759/cureus.83815

**Published:** 2025-05-09

**Authors:** Tarang Preet Kaur, Kimberly Lim Xinyi, Chloe Barnes, Shatha Attarbashi, Naweed Shahid

**Affiliations:** 1 Obstetrics and Gynaecology, Glasgow Royal Infirmary/ NHS Greater Glasgow and Clyde, Glasgow, GBR; 2 Obstetrics and Gynaecology, Saint Mary's Hospital/ Manchester University NHS Foundation Trust, Manchester, GBR; 3 Obstetrics and Gynaecology, Royal Albert Edward Infirmary/ Wrightington, Wigan and Leigh NHS Foundation Trust, Wigan, GBR

**Keywords:** autologous fascial sling, bladder neck injection, colposuspension, mesh pause, stress urinary incontinence, surgical outcomes, urogynaecology audit

## Abstract

Stress urinary incontinence (SUI) significantly impairs quality of life. This retrospective audit aimed to evaluate surgical treatment patterns and outcomes for SUI at a district general hospital between 2018 and 2023 and to compare them with national benchmarks from the third British Society of Urogynaecology (BSUG) report in 2020-21. Data from 99 patients were collected from the BSUG database and hospital electronic records, following clinical audit department approval. The mean age of patients undergoing surgery was 50 years. In 2018, mid-urethral tape was the most commonly performed procedure (46.2%); however, following the UK-wide suspension of vaginal mesh procedures that year, there was a notable shift towards alternative surgeries. Bladder neck injections (BNIs), colposuspension (open and laparoscopic), and autologous fascial sling (AFS) procedures all increased in frequency, mirroring national trends. BNIs became the predominant procedure, accounting for 76.8% of cases, with over 80% performed on an outpatient basis. Reported cure rates, when compared to national figures, were AFS (100% vs. 94%), laparoscopic colposuspension (71.4% vs. 84%), BNIs (66% vs. 60%), and open colposuspension (33.3% vs. 81%). Bladder injuries occurred in 17.3% of colposuspension cases, significantly higher than the national average of 2.7%. Additionally, prolonged catheterisation (>10 days) was noted in 29% of colposuspension cases, compared to 7.1% nationally. These elevated complication rates may reflect the procedural learning curve, as laparoscopic colposuspension was only recently introduced at the center. The audit highlights evolving surgical trends and outcomes in the management of SUI following the national mesh pause, with a shift toward non-mesh alternatives. Further audits with long-term follow-up and larger sample sizes are recommended to assess the safety, efficacy, and patient-reported outcomes of these procedures.

## Introduction

Stress urinary incontinence (SUI) is defined as an unintentional leakage of urine during activities that increase intra-abdominal pressure, such as coughing, sneezing, or physical exertion. It is a prevalent condition, affecting approximately 25-40% of women, yet only a minority seek medical care, often due to stigma or embarrassment [1,2]. SUI has a substantial impact on quality of life and carries a significant economic burden, both personally and societally. In the United Kingdom (UK), the estimated annual cost per individual ranged from £117 to £818 during the 1999-2000 financial year, though more recent national estimates are lacking [3,4].

Common risk factors for SUI include obesity, vaginal childbirth, menopause, chronic cough, and a history of pelvic surgery [1]. The underlying pathophysiology typically involves urethral hypermobility or intrinsic sphincter deficiency [5]. Initial management includes lifestyle modifications such as weight reduction, caffeine limitation, pelvic floor muscle training, and use of vaginal devices like pessaries. Surgery is considered when these measures fail. According to the National Institute for Health and Care Excellence (NICE), duloxetine may be considered in women who decline surgery or for whom conservative measures have failed [6].

Over the past few decades, surgical management of SUI has evolved significantly. Burch colposuspension was long considered the gold standard until mid-urethral sling (MUS) procedures, including transobturator tape (TOT) and single-incision mini-slings, gained popularity for being less invasive and offering favourable outcomes [7-11]. However, growing concerns about mesh-related complications prompted regulatory scrutiny, culminating in the suspension of vaginal mesh procedures in the UK in 2018 [12]. This led to a resurgence in alternative options, such as bladder neck injections (BNIs), autologous fascial sling (AFS), and both open and laparoscopic colposuspension. The COVID-19 pandemic further influenced surgical practices, resulting in a significant decline in elective continence surgeries nationwide [13].

This study aimed to audit surgical interventions for SUI over six years at a district general hospital in the National Health Service (NHS) England. It evaluated patient demographics, surgical trends, complications, and outcomes and compared findings with national data published by the British Society of Urogynaecology (BSUG) [13]. This analysis offers valuable insight into the shifting patterns in SUI management following the pause on the use of transvaginal surgical mesh and during the post-pandemic recovery period.

## Materials and methods

This retrospective audit was conducted at a District General Hospital within NHS England and was approved by the Clinical Audit and Effectiveness Department. The audit aimed to evaluate patient profiles, treatment outcomes, and surgical trends in stress urinary incontinence (SUI) procedures performed between 2018 and 2023. The results were benchmarked against national standards outlined in the 2020-2021 BSUG report [13].

Study objectives

The primary objectives were to assess the clinico-demographic characteristics of patients undergoing SUI surgery, surgical complications, patient-reported outcomes, and how local findings compared with national BSUG data from 2020-2021. The secondary objective was to evaluate temporal trends in surgical practice following the 2018 national suspension of mesh procedures and to assess the subsequent impact of the COVID-19 pandemic.

Study population and data collection

A sample size calculation was not required, as all eligible patients treated during the defined period were included. Repeat procedures for BNIs were regarded as part of the initial treatment course and were not counted as separate cases. Data were collected from theatre records and the BSUG national database. BSUG is a UK-wide online platform where consultants are routinely encouraged to record procedural and outcome data for urogynaecological surgeries after obtaining informed consent from the patient. Where entries were found incomplete, the data were supplemented from hospital electronic medical records to maximise completeness.

A structured proforma was developed based on the BSUG database to ensure comprehensive data collection. The following data points were recorded: demographics, which included age, body mass index (BMI), parity, presence of associated prolapse, and whether the surgery was primary or repeat procedure; preoperative assessment, which covered urodynamics, pelvic floor muscle training, and multidisciplinary team (MDT) discussions; surgical details, which included the type of procedure such as BNIs, AFS, open or laparoscopic colposuspension, and mid-urethral tapes such as TOT and transvaginal tape (TVT)). It also procured perioperative complications which included details such as ureteric injury, bladder injury, bowel injury, vaginal button-hole, urethral injury, neurological injury, blood loss > 500 ml, blood transfusion, thromboembolism and death. All the postoperative complications were additionally recorded under the major headings such as return to theatre within 72 hours for a procedure-related event, more than 10 days need for a catheter, return within 30 days to hospital for an event related to the procedure, readmitted to hospital within 30 days for a procedure-related event. Further information was obtained to include the reasons/explanations for the respective complications, hospital length of stay, follow-up intervals, and patient-reported outcomes, including the Patient Global Impression of Improvement (PGI-I) and changes in urgency symptoms.

Ethics

This audit was registered and approved by the NHS Trust's Quality Improvement and Audit Department. A review by the Trust's Research Committee and further confirmation via the “Is My Study Research?” tool (hra-decisiontools.org.uk) indicated that formal NHS Research Ethics Committee approval was unnecessary. Informed written consent was not required because data entry into the BSUG system is part of standard practice and consent for data recording is routinely obtained at the time of care. All data were entered into a password-protected Excel spreadsheet on a secure Trust computer. The only patient identifier used was the hospital number, which was necessary for tracking follow-up and readmissions.

Statistical analysis

Data analysis was performed using Microsoft Excel (Microsoft Corporation, Redmond, USA). Quantitative variables, such as age, BMI, length of stay, and follow-up interval, were summarised using means or medians with corresponding range values. Qualitative variables, such as parity, type of surgery, complication rates, preoperative assessments, and patient-reported outcomes, were expressed as proportions. Visual tools, including bar charts and pie charts, were used to enhance the presentation of the findings. Wherever applicable, local outcomes were compared to BSUG standards and highlighted in the data visualisations.

## Results

Patient profile

This study included a total of 99 patients who underwent surgery for SUI between 2018 and 2023. The average age of the patients was 50 years. The most frequently represented age groups were 41-50 years, comprising 30% of patients, and 51-60 years, accounting for 29%. Additional demographic data are summarised in Table 1.

**Table 1:** Baseline characteristics of patients undergoing SUI surgery (2018-23) SUI: Stress urinary incontinence

Variable	Category	Number of Patients (N=99)	Percentage (%)
Age (years)			
	31-40	22	22.2
	41-50	30	30.3
	51-60	29	29.3
	61-70	10	10.1
	≥71	8	8.1
BMI (kg/m²)			
	Normal (18.5-24.9)	15	15.2
	Overweight (25–29.9)	19	19.2
	Obese (>30)	27	27.2
	Severely obese (>40)	4	4.1
	Missing data	34	34.3
Parity			
	Nulliparous	2	2.0
	Uniparous	13	13.1
	Multiparous	84	84.9
Type of prolapse			
	Isolated cystocele	8	8.0
	Isolated rectocele	7	7.1
	Multiple prolapse types	7	7.1
	No prolapse	77	77.8
Type of incontinence			
	SUI only	57	57.6
	Mixed	42	42.4
	SUI>UI	39	39.4
	SUI=UI	2	2.0
	UI>SUI	1	1.0

Pre-procedure workup for non-mesh procedures

Pelvic floor exercises (PFEs) were offered to all patients undergoing AFS, 91% of those undergoing colposuspension, 60% of TVT/TOT patients, and 94% of BNI patients. A preoperative urodynamic study was performed in 100% of patients undergoing AFS and TVT/TOT, 96% of colposuspension patients, and 97% of BNI patients. Procedure-specific information was provided to almost all the surgical patients as summarised in Table 2.

**Table 2:** Pre-procedure workup for continence surgeries (2018-23) PFE: Pelvic floor exercises; MDT: multidisciplinary team; UDS: urodynamic studies

Surgery Type		PFE Offered	Pre-op UDS Undertaken	Procedure-Specific Information Given	Pre-op MDT
AFS	Yes (N)	4	4	4	4
	No. of cases	4	4	4	4
	%	100	100	100	100
Colposuspension	Yes (N)	21	22	23	23
	No. of cases	23	23	23	23
	%	91	96	100	100
TVT/TOT	Yes (N)	4	6	6	5
	No. of cases	6	6	6	6
	%	60	100	100	80
BNI	Yes (N)	62	64	64	64
	No. of cases	66	66	66	66
	%	94	97	97	97

Surgical trends (2018-2023) and comparison with the BSUG national report (2021-22)

**Figure 1:**
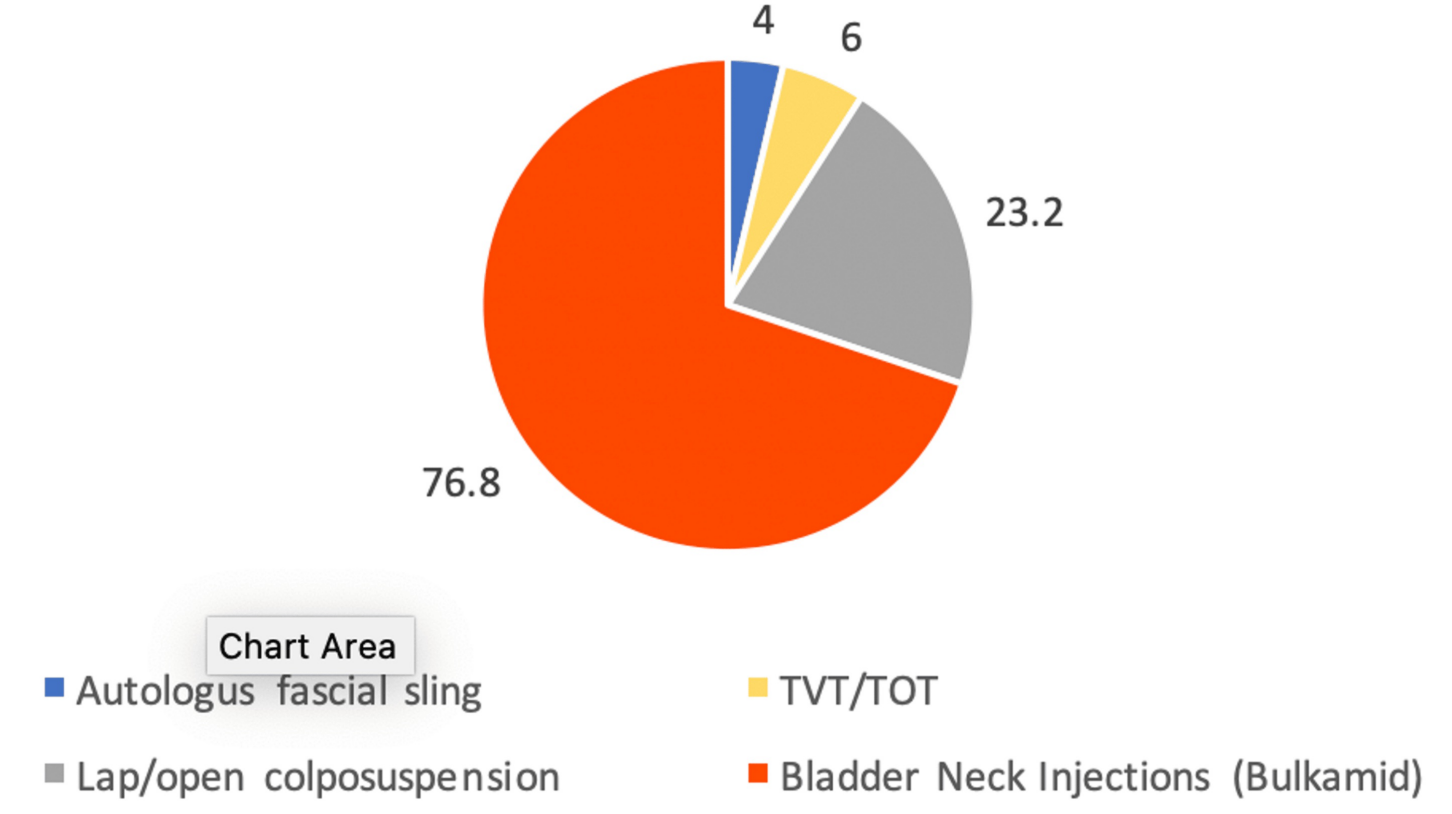
Percentage distribution of SUI surgeries (2018-23) SUI: Stress urinary incontinence; TVT: transvaginal tape; TOT: transobturator tape

From 2018 to 2023, the most commonly performed surgery at our centre was BNI (using Bulkamid), accounting for 76.8% of cases (Figure 1).

**Figure 2:**
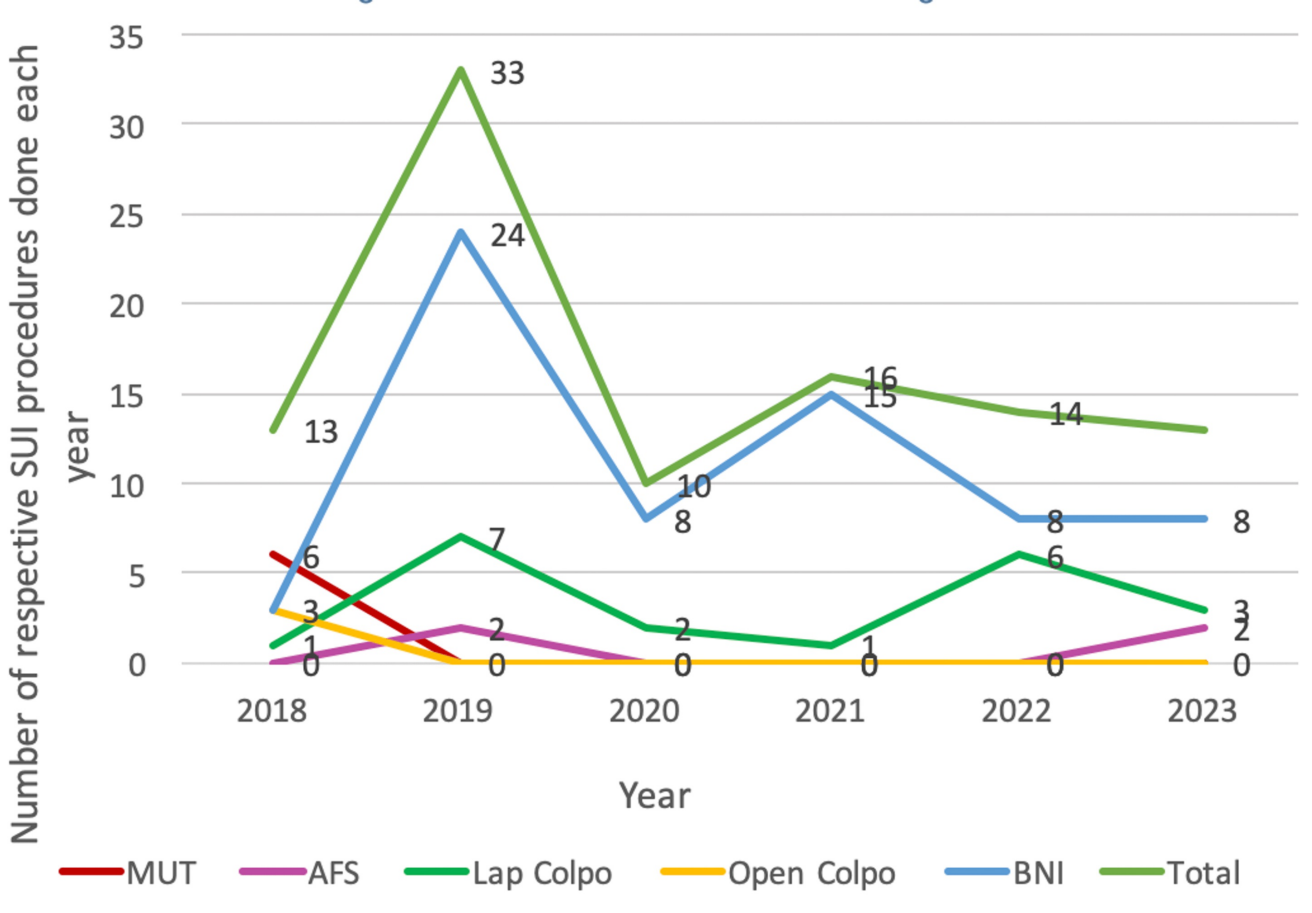
Run diagram showing a trend of continence surgeries (2018-23) in this study SUI: Stress urinary incontinence; MUT: mid-urethral tape; AFS: autologous fascial sling; BNI: bladder neck injection

In 2018, mid-urethral tape (MUT) procedures were the predominant surgical choice, representing 46.2% of all continence surgeries that year. However, following the national pause on mesh use in July 2018, there was a marked shift towards BNI, colposuspension, and AFS procedures. This shift is reflected in the annual distribution of surgical procedures illustrated in Figure 2. The COVID-19 pandemic led to a significant reduction in the number of procedures performed. Overall, the number of continence surgeries doubled following the cessation of mesh procedures but halved during the pandemic, returning to levels comparable to 2019. Since 2019, BNIs have consistently been the most commonly performed procedure annually. Trends indicate that laparoscopic colposuspension returned to pre-pandemic levels by 2022.

**Figure 3:**
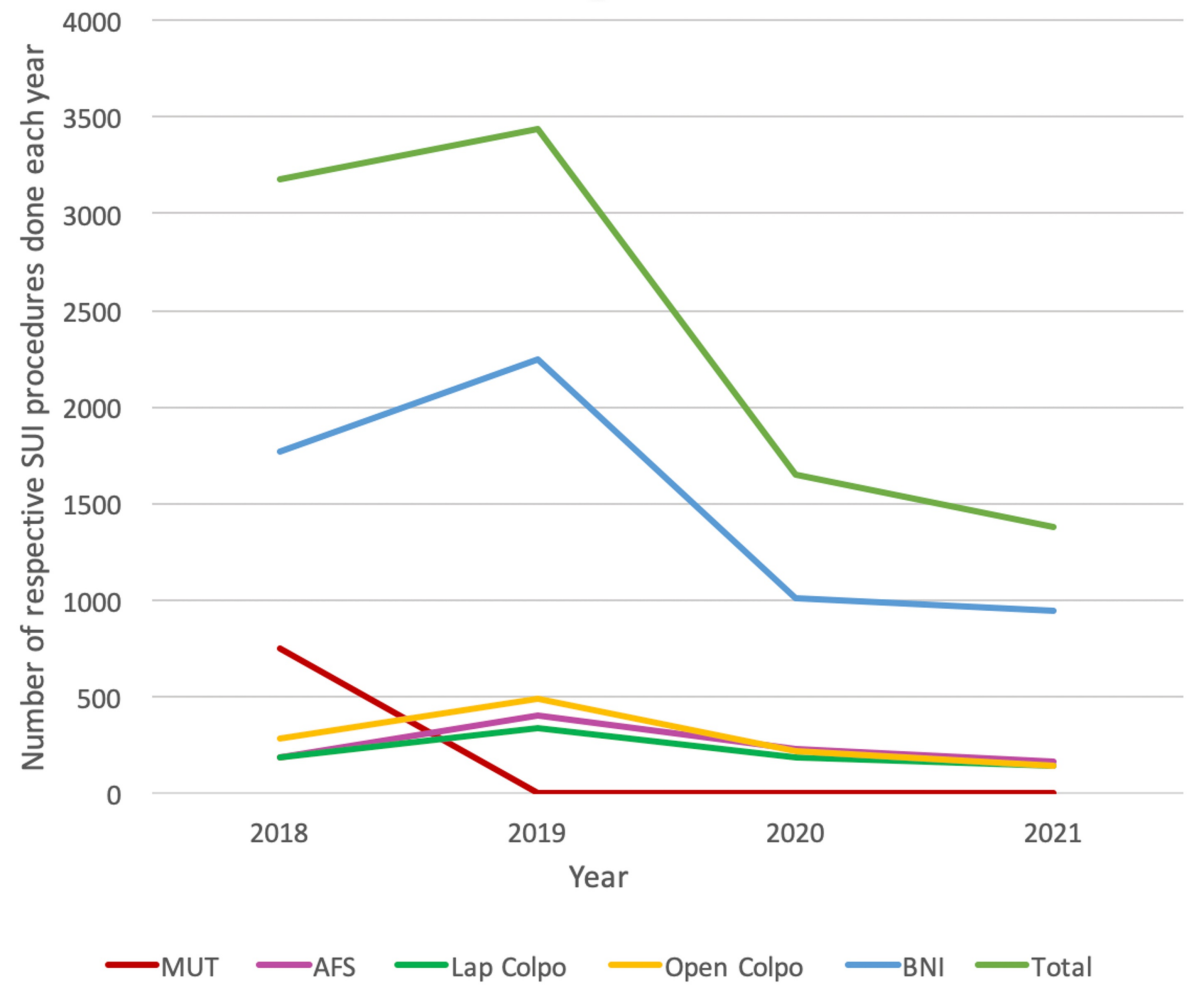
Run diagram showing the trend of continence surgeries (2018-23) in the BSUG report (2020-21) SUI: Stress urinary incontinence; MUT: mid-urethral tape; AFS: autologous fascial sling; BNI: bladder neck injection; BSUG: British Society of Urogynaecology

This pattern aligns with the BSUG national report (2018-2021), which also highlighted BNI as the most commonly performed procedure following the decline in MUT in 2018. Even during the pandemic, the BNI was performed in over 60% of cases nationally, followed by colposuspension. Nationally, AFS was performed in only a small proportion of cases, with just 6% of patients undergoing AFS in 2019. Recent trends in the BSUG report show an increase in the BNI as a primary procedure, although its use for recurrent SUI cases has declined in the post-COVID era. Conversely, the proportion of colposuspension cases remained stable between 2018 and 2021 but has increased as a choice for recurrent cases over the past two years as shown in Figure 3 [13].

Operative outcomes

In the current study, the median follow-up interval was eight weeks, compared to 12 weeks in the BSUG data. Follow-up information was available for 85% of patients against just over 50% in the BSUG report [13]. Among patients undergoing AFS, 50% attended outpatient follow-up, while all colposuspension patients (100%) had outpatient follow-up. For those who underwent BNIs, follow-up was conducted either through outpatient visits (35%) or telephone consultations (65%).

More than 80% of BNI procedures in this study were performed as outpatient cases, and less than 15% were completed as day cases, with no BNI patients requiring admission beyond 24 hours. This contrasts with national BSUG data, where only 15% of BNI procedures were performed as outpatient procedures in 2018-19, rising to 29% in 2020-21. Nationally, 64.5% of BNI patients were managed as day cases, and 6% required inpatient stays of one to four days [13].

At follow-up, prolonged catheterization was observed in 75% of AFS patients, 23.5% of colposuspension patients, 20% of TVT patients, and 1.6% of BNI patients. These rates were significantly higher compared to the national outcomes reported by BSUG [13].

Perioperative Complications

Bladder injury occurred intraoperatively in four colposuspension patients (17.3%) and one TVT patient, compared to a national bladder injury rate of 2.7% for colposuspension procedures [13]. No intraoperative injuries were reported in patients undergoing BNI or sling surgeries (Table 3).

**Table 3:** Perioperative complications of SUI surgeries and their comparison with the national BSUG report (2020-21) SUI: Stress urinary incontinence; AFS: autologous fascial sling; BNI: bladder neck injection

Perioperative Complications	AFS		Colposuspension		BNI	
	Current study	BSUG	Current study	BSUG	Current study	BSUG
Bladder injury	-	17/388 (4%)	4/23 (17.4%)	21/661 (2.7%)	-	1/1911 (0.1%)
Vaginal buttonhole	-	2/386 (0.5%)	-	-	-	-
Urethral injury	-	1/386 (0.2%)	-	-	-	-
Total	0/4		4/23 (17.4%)	-	0/66	-

Postoperative Complications

The complication profile differed significantly across procedures, with notable divergence from national benchmarks (Table 4).

**Table 4:** Postoperative complications of continence procedures and their comparison with the national BSUG report (2020-21) AFS: Autologous fascial sling; BNI: bladder neck injection; BSUG: British Society of Urogynaecology

Postoperative Complications	AFS		Colposuspension		BNI	
	Current study	BSUG	Current study	BSUG	Current study	BSUG
Return to theatre <72 hours	0/4	2/239 (1%)	0/17	3/228 (1.0%)	0/62	1/1123 (0.1%)
Catheterised >10 days	3/4(75%)	24/242 (10%)	5/17 (29%)	29/406(7.1%)	0/62	7/1122 (0.6%)
Return to hospital <30 days	1/4 (25%)	24/240 (10%)	2/17 (12%)	35/402 (8.7%)	1/62 (1.6%)	14/1119 (1%)
Readmission <30 days	1/4 (25%)	9/241 (4%)	2/17 (12%)	7/401 (1.7%)	1/62 (1.6%)	1/1118 (0.1%)
Long-term problem	0/4	0/4	1/17 (6%)		0/62	
Total (after excluding missing data entries)	4		17		62	

Patient Reported Outcomes

Global impression of improvement for incontinence: Patient-reported outcomes for all procedures are summarized in Table 5. "Very much better (VMB)" and "much better (MB)" scores were categorized as "Cure". The cure rates among those who answered, in descending order compared to the BSUG data, were as follows: AFS (100% vs 94%), BNI (66% vs 84%), laparoscopic colposuspension (71.4% vs 81%), and open colposuspension (33.3% vs 60%).

**Table 5:** Percentage of patients reporting their subjective response (PGI-I) after surgery AFS: Autologous fascial sling; BNI: bladder neck injection; TVT: transvaginal tape; TOT: transobturator tape

Patient Response	AFS		Open Colposuspension		Laparoscopic Coloposuspension		TOT		TVT		BNI	
		% (N=3)		% (N=3)		% (N=14)		% (N=1)		% (N=5)		% (N=62)
Unanswered	1	-	0		6	-	0	-	0	-	4	-
Very much better	2	75	1	33.3	10	71.4	1	100	4	80	21	33.9
Much better	1	25	0		0		0		1	20	20	32.3
Little better	0		2	66.6	2	14.4	0		0		11	17.7
No change	0		0		1	7.1	0		0		9	14.5
Little worse	0		0		1	7.1	0		0		1	1.6
Much worse	0		0		0		0		0		0	
Very much worse	0		0		0		0		0		0	
Total	4		3		20		1		5		66	
Very much better+ Much better	3	100	3	33.3	12	71.4	1	100	5	100	41	66.2

Change in urgency: Improvement or resolution of urgency was reported in 66.6% of patients who underwent AFS, and 17.6% in the colposuspension cohort. Notably, no AFS patients reported worsening or new-onset urgency, whereas 29.2% of colposuspension patients experienced worsening or new urgency (Table 6). According to BSUG data, 20-34% of patients undergoing colposuspension or fascial sling surgeries reported improved or resolved urgency, while 9-15% reported worsening or new urgency. At our centre, 16.1% of BNI patients reported improved or resolved urgency, a rate comparable to the national average of 17%. Additionally, only 1.6% of BNI patients experienced worsening or new urgency, compared to 5% nationally [13].

**Table 6:** Change in urgency in patients who underwent continence surgeries (2018-23) AFS: Autologous fascial sling; TVT: transvaginal tape; TOT: transobturator tape

Change in Urgency	AFS N=3 (Answered)	Colposuspension N=17 (Answered)	TOT N=1 (Answered)	TVT N=5 (Answered)	BNI N=62 (answered)
Cured	1 (33.3%)	0	0	2 (40%)	2 (3.2%)
Improved	1 (33.3%)	3 (17.6%)	1 (100%)	2 (40%)	8 (12.9%)
Never present	1 (33.3%)	6 (35.3%)	0	1 (20%)	37 (59.6%)
No change	0	3 (17.6%)	0	0	14 (22.6%)
New symptom	0	1 (5.8%)	0	0	0
Worse	0	4 (23.5%)	0	0	1 (1.6%)

Persistent pain at follow-up: Persistent pain was reported by 13% of colposuspension patients and 1.5% of BNI patients, with no persistent pain in patients with AFS or TOT procedures. Nationally, 9% of AFS and 1% of BNI patients reported persistent pain [13].

## Discussion

Surgical treatment provides the most effective relief in women with bothersome SUI symptoms. Previously, MUTs were the most commonly performed procedure. However, after the introduction of the warning on the use of mesh by the Food and Drug Administration (FDA), there has been a notable change in the surgical approaches for treating SUI [14]. This audit aimed to assess the quality of care provided at our centre by analysing patient profiles and surgical outcomes. This study compared the outcomes with the national BSUG report in 2020-21, taking it as the standard [13].

This study analysed the practice over six years (2018-2023) at a district general hospital. A total of 99 patients were operated to alleviate the symptoms of stress incontinence. The main focus in this study was on AFS, colposuspension and BNI due to the mesh pause in 2018. The mean age of the patients in this study was 50 years, which is in line with the median age (56 years) in the systematic review, which included 37 randomised controlled trials, 5720 participants who underwent surgical treatment for SUI [7]. However, in another study, the mean age was higher (65.7 years), and it included 1200 participants who underwent Bulkamid treatment for SUI [15].

It is essential to identify the associated urge incontinence in patients with SUI planned for surgical management. Patients with mixed urinary incontinence need appropriate counselling regarding a higher risk of surgical failure as compared to patients with pure SUI [16]. In the current study, 42% had mixed urinary incontinence.

The current study demonstrated high compliance in the pre-procedure workup, including offering PFEs, conducting urodynamic studies, providing procedure-specific patient information leaflets, and discussing cases at multidisciplinary team meetings (90-100% for AFS, colposuspension, and BNI). It resonated with the national data findings and recommended practices in the BSUG report [13]. The high compliance can be expected to contribute to enhanced patient care and outcomes, without implying a direct causal relationship that was not part of our study.

The safety concerns surrounding urogynaecological mesh implants gained significant attention following reports from the US FDA (2008, 2011), the European Commission (SCENIHR, 2015), and extensive media coverage. This led to increased litigation and debates over mid-urethral slings (MUS), ultimately resulting in the suspension of vaginal mesh surgeries in England from July 2018 [17]. The BSUG report highlighted an increasing trend in alternative continence procedures post-mesh suspension, particularly the rise of BNIs. The use of AFS and Burch colposuspension doubled in 2018-2019. The COVID-19 pandemic further impacted surgical trends, causing a 50% reduction in continence procedures, with a continued decline in 2021, though the proportion of each non-mesh procedure remained stable compared to 2019 [13]. 

In the current study, surgical trends mirrored national patterns. The BNI emerged as the most commonly performed continence procedure post-2018, followed by a 2-3 fold increase in fascial sling and laparoscopic colposuspension. The latter was introduced at this center in 2018, initially performed in collaboration with a visiting surgeon and independently by Trust consultants since 2022. Post-pandemic, laparoscopic colposuspension returned to pre-COVID levels in 2022, and by 2023, the most common procedures performed were BNI, laparoscopic colposuspension, and AFS. These findings also align with a 2021 survey-based study in Ireland, which reported a modest increase in bulking agents, with 43% of consultants and urology trainees frequently performing this procedure, compared to 29% pre-mesh pause [18]. Additionally, a study by Schimf et al. highlighted a renewed interest in pubovaginal slings, citing fewer pain-related complications and a lower risk of mesh erosion [19].

In this study, 85% of patients had documented follow-up with a median interval of 8 weeks, shorter than the 12-week interval reported in national BSUG data [13]. The majority (80%) of BNI procedures were performed on an outpatient basis, contrasting with national trend of day case procedures, and offering advantages such as reduced hospital bed occupancy and faster recovery [13].

Prolonged catheterisation (>10 days) was significantly more common following AFS and colposuspension procedures compared to national rates (75% vs 10% for AFS, and one-third vs 7.1% for colposuspension) [13]. These elevated rates may reflect the need for technique refinement. It is also plausible that the small sample size in the AFS and open colposuspension groups may have disproportionately influenced the complication rate, amplifying individual outcomes. Furthermore, it is important to note that only 50% of patients in the AFS cohort were followed up, which may have contributed to the variability in the results. In contrast, BNI showed no cases of prolonged catheterisation, which is consistent with its minimally invasive profile and aligns well with national data. Bladder injury rates were notably higher in colposuspension (17.4%) compared to the national average (2.7%), likely reflecting the early experience with laparoscopic techniques. No intraoperative complications occurred in AFS and BNI procedures in this cohort [13].

Readmission rates were higher than national figures for AFS (25% vs 4%) and colposuspension (12% vs 1.7%), with causes including hematoma, infection, and nerve entrapment. BNI showed a low readmission rate (1.6%), slightly above the national rate (0.1%). Postoperative urgency symptoms were not seen in AFS patients (vs 14.5% nationally) which could again be contributed to the lower follow-up rate and small sample size, while colposuspension showed a higher incidence of new or worsening urgency (29.2% vs 10-15%). BNI outcomes were comparable to national data [13]. A systematic review by Capobianco et al. reported a low overall rate of adverse events at 0.4%, which included worsening urinary incontinence, retention, and urgency. However, infections and urinary tract infections were notably higher, occurring in 62.5% of cases [20]. These findings must be interpreted with caution due to the limitations of a small sample size and limited follow-up, particularly in the AFS cohort. The small sample sizes may have skewed the complication rates, and the reduced follow-up could have led to an underestimation or overestimation of certain postoperative outcomes. Nevertheless, the data highlights the importance of surgical expertise with the aim of minimizing complication rates.

At a median follow-up of eight weeks, persistent pain was reported in 13% of colposuspension patients, 1.5% of BNI patients and none of the patients with AFS reported it. It is similar to the BSUG audit report, which observed persistent pain in 6.3% of Colposuspension patients, 1 % of BNI patients and 9% of AFS patients [13].

Patient reported global impression of improvement for incontinence (PGI-I) scale was used as a standard to report cure of symptoms, as used in the BSUG database. It helps in standardised reporting and comparison. At follow-up, the highest cure rates were observed in the AFS group at 100%, followed by laparoscopic colposuspension (71%), BNI (66%), and open colposuspension (33.3%). The observed cure rates for AFS and BNI were broadly consistent with national averages (94% and 60%, respectively) [13]. However, both laparoscopic and open colposuspension demonstrated lower cure rates compared to national benchmarks (91% for laparoscopic and 84% for open colposuspension) [13]. The lower cure rate for colposuspension may be attributed to the small sample size for open colposuspension and the learning curve associated with the recent introduction of laparoscopic colposuspension at this centre. The technique should be reviewed for colposuspension surgeries to improve the success rate. The small sample size for open colposuspension limits our ability to accurately compare its success rate. In a study by Schimpf et al., AFS demonstrated superior subjective cure rates compared to colposuspension, a finding consistent with the Cochrane review by Saraswat et al. [19,21]. A comprehensive review reported continence rates for open Burch procedures to be 85% at one year postoperatively and around 70% after five years, which are higher than those seen in this study at short-term follow-up [22]. Long-term data were not assessed in the current study.

An extensive systematic review analysing 175 randomised controlled trials involving 21,598 women reported the highest cure rates with traditional slings (89.4%) and open colposuspension (76.7%) [2]. These findings provide a useful comparison when interpreting the outcomes of this audit, where the cure rate for autologous fascial sling was 100% and for open colposuspension was notably lower at 33.3% [2]. A comprehensive review evaluating the efficacy and outcomes of bulking agents in women with mixed or stress urinary incontinence found pooled improvement rates of 46.0% for studies with follow-ups of ≤1 year and 57.0% for >1 year. The pooled cure rates were 26.0% for ≤1 year of follow-up and 21.0% for >1 year [20]. Short-term follow-up cure rates for BNI at our centre were fairly high. However, long-term follow-up is needed for accurate comparison. 

The strengths of this study include its comprehensive data collection over a six-year period, providing a robust timeline for analysis. The sample size of 99 patients from a single centre offers a detailed representation of the surgical outcomes at a district general hospital in England. Additionally, this study serves as the first audit conducted at the centre, offering valuable insights into local practices and trends and setting a foundation for future audits and improvements in patient care. Benchmarking outcomes against national data from the BSUG further strengthens the audit, allowing meaningful comparison and contextualisation of local findings within national standards.

The limitations of this study include its retrospective nature, which may introduce biases and limit the ability to establish causal relationships. Additionally, long-term follow-up data were unavailable, preventing an assessment of the sustained effectiveness of the procedures over time. Small sample size especially for the AFS cohort and loss to follow-up could have skewed the outcomes limiting the generalisability of the findings. Furthermore, the lack of availability of the BSUG National Report for 2022-23 means that an up-to-date, adequate comparison with national trends was not possible, potentially affecting the interpretation of the findings in the broader context. Furthermore, confounding factors such as treatment for urge incontinence and concomitant prolapse procedures were not analysed in the study, and their potential influence on the outcomes should be considered.

## Conclusions

In response to the suspension of mesh procedures in the UK, non-mesh continence surgeries have become increasingly prevalent with BNIs, colposuspension and AFS emerging as the main alternatives. BNIs became the most common, especially for primary cases. Although overall surgery rates rose initially, they declined during the COVID-19 pandemic. AFS achieved the highest continence cure rates among non-mesh procedures, aligning with national outcomes. Colposuspension, particularly the open approach, showed comparatively lower cure rates. While limited by the sample size, this may highlight the need for a technical review to optimise outcomes. Most BNIs were performed as outpatient procedures, offering practical benefits. These findings contribute valuable local data to the national conversation on continence surgery practices post-mesh suspension and highlight the evolving landscape of surgical management in a district general hospital setting.
